# The Role of Mesenchymal Stem/Stromal Cells and Their Extracellular Vesicles in Rheumatoid Arthritis

**DOI:** 10.31661/gmj.v13i.3075

**Published:** 2024-03-01

**Authors:** Amir Abaszadeh, Oveis Salehi, Ahmadreza Badali, Dorsa Lavanmardi, Alireza Sarlak, Azam Lotfalian, Maryam Poudineh

**Affiliations:** ^1^ School of Medicine , Ilam University, Ilam, Iran; ^2^ Tehran University of Medical Sciences, Tehran, Iran; ^3^ Tabriz University of Medical Sciences, Research Center for Connective Tissue Diseases; ^4^ Fars Stem Cell Research Institute, Shiraz, Fars, Iran; ^5^ Student Research Committee, School of Medicine, Hamadan University of Medical Sciences, Hamadan, Iran; ^6^ Department of Biology and Anatomical Sciences, Shahid Sadoughi University of Medical Sciences, Yazd, Iran; ^7^ School of Medicine, Mashhad Azad University, Mashhad, Iran

**Keywords:** Rheumatoid Arthritis, Mesenchymal Stem Cell, Extracellular Vesicle, Exosome

## Abstract

There is presently no cure for the chronic inflammatory disease rheumatoid arthritis (RA), which affects the joints and other organs. Mesenchymal stem/stromal cells (MSCs) are adult stem cells that are widely studied in inflammatory diseases. Several studies have shown the therapeutic effects of MSCs in preclinical and clinical RA. However, there are a few concerns regarding the use of MSCs, such as immune rejection and malignancy. Since MSCs exert their potential effects in a paracrine manner, more attention has been paid to their extracellular vesicles (MSC-EVs). In the current study, we reviewed the application of MSCs and their EVs in RA. A better understanding of the mechanisms driving MSC and MSC-EVs in RA offers insight into possible treatment options for RA.

## Introduction

Rheumatoid arthritis (RA) is an inflammatory and chronic autoimmune disease that affects the joints. RA estimates for prevalence and disease burden vary widely by geographic area, with generally higher estimates in industrialized countries and urban environments [1]. Different genetics and environmental factors are associated with the development of RA. Major histocompatibility complex (MHC) region genes, age, sex, smoking, and obesity are the most important factors [2]. RA is an immune-mediated disease and different immune cells, such as B- and T-cells play a crucial role in the pathogenesis of the disease [3]. Joints are typically affected by immune system malfunction, although the autoimmune process may start in other regions of the body. Inflammation in the synovium, the inner lining of the joint, is brought on by immune cells [4][5]. As a result of the joint’s increase in cells, proteins, and other things, the inflammation becomes chronic and the synovium thickens, which can cause redness, warmth, and pain [6].

Achieving clinical remission or slowing the course of the disease by reducing joint inflammation is the main goal of RA treatment. Conventional therapeutic options currently on the market include glucocorticoids and synthetic and biological disease-modifying anti-rheumatic drugs (DMARDs) [7]. Given the drawbacks of traditional RA medications, contemporary cellular therapy using mesenchymal stem/stromal cells (MSCs) can be considered an alternative therapeutic approach [8][9]. Besides, their extracellular vesicles (EVs) are another therapeutic option that is evaluated in preclinical studies [10][11]. Here, in the current study, we will review the application of MSCs and their EVs as new therapeutic candidates for RA.

1. Mesenchymal Stem/Stromal Cells

Mesenchymal stromal cells, also referred to as mesenchymal stem cells (MSCs), are adult stem cells with the ability of multilineage differentiation and self-renewal [12][13]. These cells can isolate from different tissues such as the umbilical cord (UC), bone marrow (BM), adipose (AD) tissue, placenta, and peripheral blood [14]. MSCs must meet the three minimal requirements established by the International Society for Cellular Therapy (ISCT), even though they may display varied traits based on the tissue from which they were derived. As a result of the absence of a commonly used surface marker phenotype, experts’ opinions on the specific characteristics of MSCs vary. However, all experts agreed that MSC populations exhibit plastic adherent properties and the expression of CD73, CD90, and CD105, and the lack of expression of hematopoietic markers, most notably CD3, CD14, CD19, CD34, and CD45 [15]. Additionally, in a culture setting, MSCs can differentiate into three mesodermal lineages chondrocytes, adipocytes, and osteoblasts [16]. Different studies have demonstrated the therapeutic potential of MSCs in inflammatory diseases, especially in autoimmune diseases [17]. The role of MSCs has been demonstrated in multiple sclerosis (MS) [18], RA [19], systemic lupus erythematosus (SLE) [20], graft-versus-host disease (GvHD) [21], Alzheimer’s disease (AD) [22], Parkinson’s disease (PD) [23], etc. MSCs were first believed to function therapeutically by migrating to damaged areas, engrafting, and then differentiating into the required cells for tissue regeneration. Other research, however, suggests that the therapeutic efficacy of MSCs is a result of the substances they produce as well as their differentiation [24].

2. Extracellular Vesicles

EVs are nanosized membrane-bound vesicles that range in size from 30 to 1000 nm and carry essential substances between cells, maintain physiological homeostasis, and have an impact on pathogenesis [25][26]. Exosomes (30-150 nm), microvesicles (100-1000 nm), and apoptotic bodies (50-5000 nm) are the three subgroups of EVs based on their diameter. Different cell types secrete EVs, such as dendritic cells (DCs), mast cells, B-cells, T-Cells, epithelial cells, neural cells, etc [15][27]. Recent research revealed that MSC-EVs mediate the paracrine effects of the MSCs, exert biological effects similar to those of the parent cells, and have regenerative and anti-inflammatory capabilities in animal models of MS [28], traumatic brain injury (TBI) [29], stroke [30], RA [31], AD [32], PD [33], GvHD [34], wound healing [35], perinatal brain injury [36], etc. It is also worth mentioning that MSC-EVs have some advantages in comparison to MSC therapy, including low tumorigenicity (they are not self-replicating) and immunogenicity [37], the ability to pass the blood-brain barrier (BBB) [38], the ability to encapsulate substances that can load particular drugs and can transport those substances to target cells [39], and finally, they also can preserve their internal biomolecular activity for an extended period at -80°C without deactivating [40]. Since, the therapeutic effects of MSCs and MSC-EVs have been shown in RA, in the following sections we will review the latest research on the application of MSCs and MSC-EVs on RA.

3.Application of MSCs in RA

3.1. Preclinical Studies

Many studies examined the therapeutic efficacy of MSCs in animal models of RA. Liu et al. investigated the efficacy and safety of a single dose intravenous (I.V.) infusion hUC-MSC in collagen-induced arthritis (CIA). Their results demonstrated a decrease in clinical score, histological store, and concentration of interleukin (IL)-6 [41]. One of the vital effects of MSCs that makes them suitable therapeutic candidates for RA is their immunomodulatory effects. Since RA is an autoimmune disease, most preclinical studies evaluated the immunomodulatory effects of MSCs on immune cells [42].

T cells are immune cells that play an important role in the RA’s pathogenesis. Previous studies have shown the ability of MSCs to control the activity, differentiation, and proliferation of T cells and also decrease the production of pro-inflammatory cytokines. In this regard, Yu et al. have shown that in I.V. administration of human umbilical cord blood MSCs (hUCB-MSCs) at different doses in CIA significantly improves the clinical joint score, decreases cartilage damage, joint inflammation, cartilage damage, IL-1β, and IL-6 protein level and increase the frequency of regulatory T (Treg) cells and the protein level of IL-10 [43].

In another study, Gonzalo-Gil investigated the therapeutic effects of human embryonic stem cell-derived MSCs (hESC-MSC) in the CIA model. Administration of hESC-MSC significantly reduces the severity of the disease and increases Foxp3+ Treg cells [44]. Liu et al. showed that the injection of hUC-MSCs in a mouse model of CIA delayed the onset of disease by lowering the number and activities of T follicular helper cells (Tfh) through IDO activity [45]. In a study by Vohra et al., they showed that UC-MSCs dramatically reduced the activation and proliferation of CD4+ and CD8+ T cells and induce the expansion of Treg cells in RA patients [46]. Lopez-Santalla also demonstrated that the administration of human-expanded AD-MSCs to CIA mice reduces the number of pathogenic T CD4+ GM-CSF+ cells and increases the number of Foxp3+ CD4+ Treg cells and IL10+ IL17- CD4+ T cells [47]. The role of CD8+ T cells has not been well evaluated in the pathogenesis of RA. But there is proof that the quantity of CTLs in the joint, where a high frequency of CD8+ T cells have been seen at the site of inflammation, is directly correlated with the severity of RA [48]. Macrophages are other immune cells that play a vital role in the pathogenesis of RA. Previous research has shown the presence of pro-inflammatory HLA-DR+ macrophages in in RA patients’ synovium [49]. Shin et al. have demonstrated that the administration of hUCB-MSCs into the CIA mice after the onset of the disease significantly ameliorates the severity of the disease by regulating the function of macrophages. In the study, the authors showed that cyclooxygenase-2 and TNF-stimulated gene/protein 6 activations by tumor necrosis factor (TNF)-α, polarized naive macrophages toward an M2 phenotype when macrophages were co-cultured with hUCB-MSCs [50].

In conclusion, independent of the tissue origin of the MSCs and the method of delivery, MSC-based therapy can reduce the level of arthritic inflammation in most experimental models of RA.

3.2. Clinical Studies

Clinical trials are ongoing for several MSC-based treatments for the treatment of RA. Some of these clinical trials have already been published and the others are ongoing. Most of the studies evaluated the efficacy and safety of MSCs therapy in RA patients [51].

Alvaro-Garcia et al. examined the safety and tolerability of I.V. administration of allogenic ASCs in refractory RA patients. They used different doses of ASCs and their results showed that at the dose range and period investigated, the I.V. infusion of ASCs was generally well tolerated and showed no signs of dose-related toxicity. Additionally, a trend toward clinical effectiveness was seen [52]. In another study by Shadmanfar et al., they investigated the tolerability and safety of autologous BM-MSCs I.V. administration in RA patients with knee involvement. Their results showed no adverse effect after administration and 12 months of follow-up and improvement in pain-free walking distance, time to jelling, visual analogue scale (VAS), and Western Ontario and McMaster Universities Arthritis Index (WOMAC). They also showed that methotrexate (MTX) and prednisolone usage looked to have decreased as a result of the MSCs therapy [53]. In a similar study in Iran, Ghoryani et al. investigated the effects of I.V. administration of autologous BM-MSCs in refractory RA patients. Their results showed decreasing in Th17 percentage, Disease Activity Score-28 with Erythrocyte Sedimentation Rate (DAS28-ESR), and VAS score [54]. UC-MSCs have also been used in clinical trials of RA. In this regard, Wang et al. demonstrated that the I.V. administration of DMARDS plus UC-MSCs significantly decreases the serum level of TNF-α and IL-6, increases the frequency of Treg cells, significant remission of disease, and improvement in DAS28 [55]. The long-term assessment also showed no abnormalities in the kidney and liver function, blood routine, and immunoglobin examination. Also, the ESR, C-reactive protein (CRP), rheumatoid factor (RF), and anti-cyclic citrullinated Peptide (CCP) after one and three years post-treatment were found to be lower than those at pre-treatment, demonstrating a significant reduction. One year and three years after therapy, the health index (HAQ) and joint function index (DAS28) were lower than they were before treatment [56].

In another similar study by Park et al., they showed that the administration of hUC-MSCs to RA patients had no major toxicity and decrease the level of pro-inflammatory cytokines including IL-8, IL-1β, IL-6, and TNF-α [57].

In conclusion, in any of the RA clinical trials that have been conducted, no toxicity or negative effects have been identified. The fact that the majority of the RA patients participating in these studies were refractory to standard RA medications and had a long history of the disease likely contributed to the lack of sufficient data on efficacy.

4. Application of MSC-EVs in RA

Immune rejection, genetic disabilities, and malignancy are perennial worries in cell treatment. Since EVs therapy is spontaneously secreted and widely disseminated in bodily fluids, it has better treatment outcomes than stem cell therapy and is also well tolerated by the body. They offer cell-free therapeutic applications and may be properly preserved while minimizing the dangers of malignant transformation, immunological rejection, and occlusion of tiny vessels associated with cell treatment [15]. Many studies have shown the therapeutic effects of MSC-EVs in RA. Tian et al. have investigated the therapeutic effects of human gingival mesenchymal stem cells-derived exosome (GMSC-Exo) in CIA animal models. Their results showed that GMSC-Exo inhibits IL-17A and promotes IL-10, decreasing incidences and bone erosion of arthritis, via inhibiting IL-17RA-Act1-TRAF6-NF-κB signal pathway similar or stronger than GMSCs [58]. Chen et al. also evaluated the therapeutic effects of bone-marrow MSC-derived miR-150-5p exosomes (Exo-150) on joint destruction in the CIA animal model. Their results showed that by targeting Matrix Metallopeptidase 14 (MMP14) and Vascular endothelial growth factor (VEGF), Exo-150 downregulated tube formation in HUVECs and reduced migration and invasion in RA Fibroblast-like synoviocytes (FLS). Exo-150 injection in mice with CIA decreased the thickness of the hind paws and the clinical arthritic scores. Exo-150 decreased joint degeneration by preventing angiogenesis and synoviocyte hyperplasia. Exosomes are a potential RA treatment method because they make direct intracellular miRNA transfer between cells possible [59]. In another study by Xu et al., they demonstrated the administration effect of hUC-MSC-derived sEVs on the CIA animal model. Their results showed that in a dose-dependent way, sEV therapy reduced arthritic symptoms and prevented synovial hyperplasia. These benefits were achieved by reducing the number of Th17 cells and increasing the fraction of Treg cells in the spleen, which decreased serum IL-17 and increased IL-10 and TGF-β expression while enhancing T lymphocyte apoptosis. In terms of transcription, sEVs boosted Foxp3 expression and decreased RORγt expression in the spleen while increasing Foxp3 expression in the joints. In certain ways, sEVs were better at treating CIA than MSCs and MTX [60].

## Conclusion

Treatment for RA is challenging. The existing RA treatment techniques may not be able to relieve RA symptoms in all patients. MSCs and their EVs are promising therapeutic options that have been evaluated in different studies. It’s an intriguing idea that MSCs may be able to reprogram an autoimmune process into a more naive, tolerant state. To maximize the therapeutic potential of MSCs, research into the shared microenvironment and how it interacts with the supplied cell types is essential. In parallel, various preclinical models are being used to assess the therapeutic use of EVs recovered from various cells. Even if there is a lot of evidence of the effectiveness of EVs produced from MSCs in numerous animal models, it is still insufficient for the treatment of rheumatic disease. However, the expanding volume of studies on MSC-EVs will probably help in better defining treatment alternatives. To comprehend the mechanisms underlying the therapeutic effect of MSC-EVs, additional research on these cells is necessary.

## Conflict of Interest

The authors declare that there is no conflict of interest regarding the publication of this article.

## References

[R1] Finckh A, Gilbert B, Hodkinson B, Bae S-C, Thomas R, Deane KD (2022). Global epidemiology of rheumatoid arthritis. Nat Rev Rheumatol.

[R2] Deane KD, Demoruelle MK, Kelmenson LB, Kuhn KA, Norris JM, Holers VM (2017). Genetic and environmental risk factors for rheumatoid arthritis. Best Pract Res Clin Rheumatol.

[R3] Jang S, Kwon E-J, Lee JJ (2022). Rheumatoid arthritis: pathogenic roles of diverse immune cells. Int J Mol Sci.

[R4] Komatsu N, Takayanagi H (2022). Mechanisms of joint destruction in rheumatoid arthritis—immune
cell–fibroblast–bone interactions. Nat Rev Rheumatol.

[R5] Adugani S, Bannimath G, Sastry P (2021). A review on biomarkers in clinical osteoporosis-Significance of
hydroxyproline. Biomed Biotechnol Res J.

[R6] McInnes IB, Schett G (2011). The pathogenesis of rheumatoid arthritis. N Engl J Med.

[R7] Guo Q, Wang Y, Xu D, Nossent J, Pavlos NJ, Xu J (2018). Rheumatoid arthritis: pathological mechanisms and modern
pharmacologic therapies. Bone Res.

[R8] Lopez-Santalla M, Bueren JA, Garin MI (2021). Mesenchymal stem/stromal cell-based therapy for the treatment of
rheumatoid arthritis: An update on preclinical studies. EBioMedicine.

[R9] Brown C, McKee C, Bakshi S, Walker K, Hakman E, Halassy S, Svinarich D, Dodds R, Govind CK, Chaudhry GR (2019). Mesenchymal stem cells: Cell therapy and regeneration potential. Journal of tissue engineering and regenerative medicine.

[R10] Cosenza S, Ruiz M, Maumus M, Jorgensen C, Noël D (2017). Pathogenic or therapeutic extracellular vesicles in rheumatic
diseases: role of mesenchymal stem cell-derived vesicles. Int J Mol Sci.

[R11] Shaik M, Shaik M, Subramanyam G, Rajasekhar G (2021). Identification of a subpopulation of chemoresistant cancer cells
with adult stem cell properties and embryonic transcription factors in oral
squamous cell carcinoma. Biomedical and Biotechnology Research Journal.

[R12] Jiang Y, Jahagirdar BN, Reinhardt RL, Schwartz RE, Keene CD, Ortiz-Gonzalez XR (2002). Pluripotency of mesenchymal stem cells derived from adult
marrow. Nature.

[R13] Za'ror YSMA, Zulkafli Z, Al-Eitan LN, Elsalem L, Al-Husein BA, Azlan M (2022). The Expression of BCL11A, KLF1, and ERK of Mitogen-Activated
Protein Kinase Pathway on Stem Cell Factor and Erythropoietin-Treated K562
Cells. Biomed Biotechnol Res J.

[R14] Meirelles LdS, Chagastelles PC, Nardi NB (2006). Mesenchymal stem cells reside in virtually all post-natal organs
and tissues. J Cell Sci.

[R15] Jafarinia M, Alsahebfosoul F, Salehi H, Eskandari N, Ganjalikhani-Hakemi M (2020). Mesenchymal stem cell-derived extracellular vesicles: a novel
cell-free therapy. Immunol Invest.

[R16] Azimzadeh M, Mahmoodi M, Kazemi M, Hakemi MG, Jafarinia M, Eslami A (2020). The immunoregulatory and neuroprotective effects of human adipose
derived stem cells overexpressing IL-11 and IL-13 in the experimental
autoimmune encephalomyelitis mice. Int Immunopharmacol.

[R17] DelaRosa O, Dalemans W, Lombardo E (2012). Mesenchymal stem cells as therapeutic agents of inflammatory and
autoimmune diseases. Curr Opin Biotechnol.

[R18] Mansoor SR, Zabihi E, Ghasemi-Kasman M (2019). The potential use of mesenchymal stem cells for the treatment of
multiple sclerosis. Life Sci.

[R19] Wang Y, Ma D, Wu Z, Yang B, Li R, Zhao X (2021). Clinical application of mesenchymal stem cells in rheumatic
diseases. Stem Cell Res Ther.

[R20] Tang W-Y, Liu J-H, Peng C-J, Liao Y, Luo J-S, Sun X (2021). Functional characteristics and application of mesenchymal stem
cells in systemic lupus erythematosus. Arch Immunol Ther Exp (Warsz).

[R21] Elgaz S, Kuçi Z, Kuçi S, Bönig H, Bader P (2019). Clinical use of mesenchymal stromal cells in the treatment of
acute graft-versus-host disease. Transfus Med Hemother.

[R22] Chakari-Khiavi F, Dolati S, Chakari-Khiavi A, Abbaszadeh H, Aghebati-Maleki L, Pourlak T (2019). Prospects for the application of mesenchymal stem cells in
Alzheimer's disease treatment. Life Sci.

[R23] Heris RM, Shirvaliloo M, Abbaspour-Aghdam S, Hazrati A, Shariati A, Youshanlouei HR (2022). The potential use of mesenchymal stem cells and their exosomes in
Parkinson’s disease treatment. Stem Cell Res Ther.

[R24] Madrigal M, Rao KS, Riordan NH (2014). A review of therapeutic effects of mesenchymal stem cell
secretions and induction of secretory modification by different culture
methods. J Transl Med.

[R25] Tetta C, Ghigo E, Silengo L, Deregibus MC, Camussi G (2013). Extracellular vesicles as an emerging mechanism of cell-to-cell
communication. Endocrine.

[R26] Bharathy MS, Jeyaleela GD, Vimala JDR, Agila A, Sheela SAM (2021). Comparison of anti-inflammatory activities of biogenic Gymnema
sylvestre-and Panicum sumatrense-mediated titanium dioxide nanoparticles. Biomedical and Biotechnology Research Journal (BBRJ).

[R27] Agila A, Vimala JDR, Bharathy MS, Jeyaleela GD, Sheela SAM (2022). Anti-Oxidant and anti-cancer activities of biogenic synthesized
copper oxide nanoparticles. Biomedical and Biotechnology Research Journal (BBRJ).

[R28] Jafarinia M, Alsahebfosoul F, Salehi H, Eskandari N, Azimzadeh M, Mahmoodi M (2020). Therapeutic effects of extracellular vesicles from human
adipose-derived mesenchymal stem cells on chronic experimental autoimmune
encephalomyelitis. J Cell Physiol.

[R29] Muhammad SA (2019). Mesenchymal stromal cell secretome as a therapeutic strategy for
traumatic brain injury. Biofactors.

[R30] Bang OY, Kim EH (2019). Mesenchymal stem cell-derived extracellular vesicle therapy for
stroke: challenges and progress. Front Neurol.

[R31] Liu H, Li R, Liu T, Yang L, Yin G, Xie Q (2020). Immunomodulatory effects of mesenchymal stem cells and
mesenchymal stem cell-derived extracellular vesicles in rheumatoid arthritis. Front Immunol.

[R32] Cone AS, Yuan X, Sun L, Duke LC, Vreones MP, Carrier AN (2021). Mesenchymal stem cell-derived extracellular vesicles ameliorate
Alzheimer's disease-like phenotypes in a preclinical mouse model. Theranostics.

[R33] Herman S, Fishel I, Offen D (2021). Intranasal delivery of mesenchymal stem cells-derived
extracellular vesicles for the treatment of neurological diseases. Stem Cells.

[R34] Fujii S, Miura Y, Fujishiro A, Shindo T, Shimazu Y, Hirai H (2018). Graft-versus-host disease amelioration by human bone marrow
mesenchymal stromal/stem cell-derived extracellular vesicles is associated
with peripheral preservation of naive T cell populations. Stem Cells.

[R35] Casado-Díaz A, Quesada-Gómez JM, Dorado G (2020). Extracellular vesicles derived from mesenchymal stem cells (MSC)
in regenerative medicine: applications in skin wound healing. Front Bioeng Biotechnol.

[R36] Sisa C, Kholia S, Naylor J, Herrera Sanchez, Bruno S, Deregibus MC (2019). Mesenchymal stromal cell derived extracellular vesicles reduce
hypoxia-ischaemia induced perinatal brain injury. Front Physiol.

[R37] Phinney DG, Pittenger MF (2017). Concise review: MSC-derived exosomes for cell-free therapy. Stem Cells.

[R38] Milbank E, Dragano NR, González-García I, Garcia MR, Rivas-Limeres V, Perdomo L (2021). Small extracellular vesicle-mediated targeting of hypothalamic
AMPKα1 corrects obesity through BAT activation. Nat Metab.

[R39] Herrmann IK, Wood MJA, Fuhrmann G (2021). Extracellular vesicles as a next-generation drug delivery
platform. Nat Nanotechnol.

[R40] Watanabe Y, Tsuchiya A, Terai S (2021). The development of mesenchymal stem cell therapy in the present,
and the perspective of cell-free therapy in the future. Clin Mol Hepatol.

[R41] Liu L, Farhoodi HP, Han M, Liu G, Yu J, Nguyen L (2020). Preclinical evaluation of a single intravenous infusion of
hUC-MSC (BX-U001) in rheumatoid arthritis. Cell Transplant.

[R42] Shandil RK, Dhup S, Narayanan S (2022). Evaluation of the therapeutic potential of mesenchymal stem cells
(MSCs) in preclinical models of autoimmune diseases. Stem Cells Int.

[R43] Yu Y, Yoon KA, Kang TW, Jeon HJ, Sim YB, Choe SH (2019). Therapeutic effect of long-interval repeated intravenous
administration of human umbilical cord blood-derived mesenchymal stem cells
in DBA/1 mice with collagen-induced arthritis. J Tissue Eng Regen Med.

[R44] Gonzalo-Gil E, Pérez-Lorenzo MJ, Galindo M, Díaz de, López-Millán B, Bueno C (2016). Human embryonic stem cell-derived mesenchymal stromal cells
ameliorate collagen-induced arthritis by inducing host-derived indoleamine
2, 3 dioxygenase. Arthritis Res Ther.

[R45] Liu R, Li X, Zhang Z, Zhou M, Sun Y, Su D (2015). Allogeneic mesenchymal stem cells inhibited T follicular helper
cell generation in rheumatoid arthritis. Sci Rep.

[R46] Vohra M, Sharma A, Bagga R, Arora SK (2020). Human umbilical cord-derived mesenchymal stem cells induce tissue
repair and regeneration in collagen-induced arthritis in rats. J Clin Transl Res.

[R47] Lopez-Santalla M, Mancheño-Corvo P, Menta R, Lopez-Belmonte J, DelaRosa O, Bueren JA (2015). Human adipose-derived mesenchymal stem cells modulate
experimental autoimmune arthritis by modifying early adaptive T cell
responses. Stem Cells.

[R48] Petrelli A, Van Wijk (2016). CD8+ T cells in human autoimmune arthritis: the unusual
suspects. Nat Rev Rheumatol.

[R49] Mulherin D, Fitzgerald O, Bresnihan B (1996). Synovial tissue macrophage populations and articular damage in
rheumatoid arthritis. Arthritis Rheum.

[R50] Shin T-H, Kim H-S, Kang T-W, Lee B-C, Lee H-Y, Kim Y-J (2016). Human umbilical cord blood-stem cells direct macrophage
polarization and block inflammasome activation to alleviate rheumatoid
arthritis. Cell Death Dis.

[R51] Lopez-Santalla M, Fernandez-Perez R, Garin MI (2020). Mesenchymal stem/stromal cells for rheumatoid arthritis
treatment: an update on clinical applications. Cells.

[R52] Álvaro-Gracia JM, Jover JA, García-Vicuña R, Carreño L, Alonso A, Marsal S (2017). Intravenous administration of expanded allogeneic adipose-derived
mesenchymal stem cells in refractory rheumatoid arthritis (Cx611): results
of a multicentre, dose escalation, randomised, single-blind,
placebo-controlled phase Ib/IIa clinical trial. Ann Rheum Dis.

[R53] Shadmanfar S, Labibzadeh N, Emadedin M, Jaroughi N, Azimian V, Mardpour S (2018). Intra-articular knee implantation of autologous bone
marrow–derived mesenchymal stromal cells in rheumatoid arthritis patients
with knee involvement: Results of a randomized, triple-blind,
placebo-controlled phase 1/2 clinical trial. Cytotherapy.

[R54] Ghoryani M, Shariati-Sarabi Z, Tavakkol-Afshari J, Ghasemi A, Poursamimi J, Mohammadi M (2019). Amelioration of clinical symptoms of patients with refractory
rheumatoid arthritis following treatment with autologous bone marrow-derived
mesenchymal stem cells: a successful clinical trial in Iran. Biomed Pharmacother.

[R55] Wang L, Wang L, Cong X, Liu G, Zhou J, Bai B (2013). Human umbilical cord mesenchymal stem cell therapAbaszadeh Ay for
patients with active rheumatoid arthritis: safety and efficacy. Stem Cells Dev.

[R56] Wang L, Huang S, Li S, Li M, Shi J, Bai W (2019). Efficacy and safety of umbilical cord mesenchymal stem cell
therapy for rheumatoid arthritis patients: a prospective phase I/II study. Drug Des Devel Ther.

[R57] Park EH, Lim H-s, Lee S, Roh K, Seo K-W, Kang K-S (2018). Intravenous infusion of umbilical cord blood-derived mesenchymal
stem cells in rheumatoid arthritis: a phase Ia clinical trial. Stem Cells Transl Med.

[R58] Tian X, Wei W, Cao Y, Ao T, Huang F, Javed R (2022). Gingival mesenchymal stem cell-derived exosomes are
immunosuppressive in preventing collagen-induced arthritis. J Cell Mol Med.

[R59] Chen Z, Wang H, Xia Y, Yan F, Lu Y (2018). Therapeutic potential of mesenchymal cell–derived
miRNA-150-5p–expressing exosomes in rheumatoid arthritis mediated by the
modulation of MMP14 and VEGF. J Immunol.

[R60] Xu K, Ma D, Zhang G, Gao J, Su Y, Liu S (2021). Human umbilical cord mesenchymal stem cell-derived small
extracellular vesicles ameliorate collagen-induced arthritis via
immunomodulatory T lymphocytes. Mol Immunol.

